# Effects of Pb Smelting on the Soil Bacterial Community near a Secondary Lead Plant

**DOI:** 10.3390/ijerph15051030

**Published:** 2018-05-20

**Authors:** Zhanbin Luo, Jing Ma, Fu Chen, Xiaoxiao Li, Shaoliang Zhang

**Affiliations:** 1 School of Environment Science and Spatial Informatics, China University of Mining and Technology, Xuzhou 221008, Jiangsu, China; lzbin1991@cumt.edu.cn (Z.L.); jingma2013@cumt.edu.cn (J.M.); lixiaoxiao@cumt.edu.cn (X.L.); slzhang@cumt.edu.cn (S.Z.); 2 Low Carbon Energy Institute, China University of Mining and Technology, Xuzhou 221008, Jiangsu, China; 3 Amap, Inra, Cnrs, Ird, Cirad, University of Montpellier, 34090 Montpellier, France

**Keywords:** secondary lead, bacterial community, lead contamination, high-throughput sequencing, environmental management

## Abstract

Secondary lead smelting is a widespread industrial activity which has exacerbated Pb or Cd contamination of soil and water across the world. Soil physicochemical properties, soil enzyme activities, heavy metal concentrations, and bacterial diversity near a secondary lead plant in Xuzhou, China were examined in this study. The results showed that secondary lead smelting activities influenced nearby soils. Soil acidification decreased one order of magnitude, with a mean value of 7.3. Soil organic matter also showed a downward trend, while potassium and nitrogen appeared to accumulate. Soil urease and protease activity increased in samples with greater heavy metal pollution, but overall the soil microbial biodiversity decreased. Soil heavy metal concentration—especially Pb and Cd—greatly exceeded the concentrations of Chinese Environmental Quality Standard for Soils (GB 15618-1995). Some environmental factors—such as pH, organic matter, enzyme activity, and the concentration of heavy metals—significantly affected bacterial diversity: compared with the control site, the Chao1 estimator decreased about 50%, while the Shannon diversity index dropped approximately 20%. Moreover, some genera have significant relationships with heavy metal concentration—such as *Ramlibacter* with Zn and *Steroidobacter* with Cd—which might act as bio-indicators for soil remediation. These results will provide a new insight in the future for reclaiming soil contaminants caused by secondary lead smelting.

## 1. Introduction

Lead is an indispensable component in anti-corrosives, anti-radiation materials, and lead–acid batteries, which has played an essential role in industrial circles [1,2]. In the past decade, global lead consumption has continued to increase [3]. Due to non-renewability [4], secondary lead has been an important source [5,6]. The worldwide production of refined lead was approximately 108.9 million tonnes in 2015; more than half, about 61.7 million tonnes, came from secondary lead; and 70% of secondary lead was disassembled from waste lead–acid batteries [3,7]. Unfortunately, the disassembling processes of waste lead–acid batteries—which contains crushing, fusion, reduction, and refining—causes large quantities of smoke dust and wastewater into the surrounding soil and water bodies [8,9,10,11]. These wastes can destroy ecosystems and threaten public health [12,13].

Due to the effect of Pb on the brain and cognition of children with resulting behavioral changes, lead pollution has widely been a concern [14,15,16,17]. Lead is a toxic heavy metal that has adverse effects on human lung, kidney, and reproductive and cardiovascular systems [13]. People are exposed to lead through breathing lead-polluted air or the ingestion of lead in food and drink [4]. The main manifestation of lead poisoning is mental decline, kidney injury, infertility, abortion and hypertension [18]. It can also cause lead encephalopathy, abdominal colic, multiple neuritis, hemolytic anemia, etc. [18,19].

However, there remain some deficiencies in understanding toxicities around industries working with secondary lead. Some studies on secondary lead smelting activities have been conducted to access environmental toxicity of the by-products of secondary lead production and health risks [9,20,21,22,23]. Soil heavy metal concentrations have been reported to have significantly breached the threshold trigger values that were set in their background in the vicinity of the secondary lead plants in many countries, such as southern Sweden [9], France [11], the Czech Republic [24], China [25], and Cameroon [26]. The by-products of secondary lead smelting affect blood lead levels, cognition, anemia, and other health effects, especially for children [27,28]. Few studies have paid attention to bacterial diversity near secondary lead plants, which has an essential role in the promotion and circulation of energy and materials in soil micro-ecosystems that make them indispensable in stabilizing said ecosystems [29,30].

The aims of this study are as follows: (i) access heavy metal contamination in the vicinity of the secondary lead plant in Xuzhou, China; (ii) determine the soil bacterial community diversity using high-throughput DNA sequencing technology under different heavy metal concentrations; and (iii) explore the relationship between the environmental factors and the soil bacterial community structure and diversity. This study might help to understand the resistance of the soil bacterial community and provide new insights in the future for reclaiming soil contamination caused by secondary lead smelting. 

## 2. Materials and Methods

### 2.1. Study Area and Sample Collection

This study area was located in Xuzhou (34°18′11″ N, 118°2′17″ E) in Eastern China. The area enjoys a warm, semi-humid monsoon climate with an annual average temperature of 14.0 °C, an annual average rainfall of 867.8 mm, and an annual average of 2318.6 sunshine hours [31]. The secondary lead plant mainly uses waste lead–acid batteries as the raw material to produce fine lead and alloy lead. The annual secondary lead production is nearly 600,000 tonnes. Moreover, the annual design recovery capacity of waste lead–acid battery is about one million tonnes. It is one of the largest plants in the comprehensive utilization of waste lead–acid batteries in China [32].

**Figure 1:**
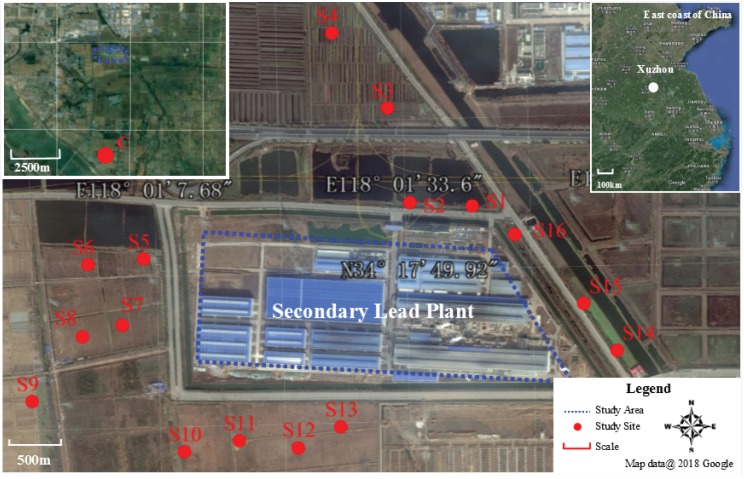
Location of the secondary lead plant and soil sampling sites.

Soil samples were collected from the 0–20 cm topsoil near the secondary lead plant in November 2016 (Figure 1). Soil sites were labeled based on the order which they were collected: S1–S16. A control sample, labelled C, was collected from a non-contamination site in 5 km away. All the 17 soil sites had the same soil type and soil texture. Each sample was collected using the plum blossom five-subsample method [33]. The five soil subsamples were crushed, mixed, and amalgamated in the field into one by sample quartering. These soil samples, approximately one kilogram, were placed and marked in labelled polyvinyl chloride bags and brought to the laboratory in a timely manner. In the laboratory, the samples were divided into three equal parts. One part was air dried, sieved through a 0.149 mm mesh, and tested for physicochemical properties and heavy metal contents. Another part was used to measure soil enzyme activity immediately in a fresh soil sample. The third was stored at −20 °C for subsequent analysis of microbial diversity [33].

### 2.2. Soil Processing and Pollution Assessment

Soil pH values were measured using a glass electrode (PHC-3C, Shanghai Leici, Shanghai, China) in a 1:5 suspension of ultrapure water [29]. Soil organic matter content was measured with the potassium chromate oxidation–colorimetric method [34]. The available phosphorus contents were determined by the hydrochloric acid ammonium chloride method [35]. The ammonium acetate extraction-colorimetric method was used to measure available potassium [33]. Soil nitrate nitrogen contents were determined by the calcium chloride–colorimetry method [36].

Soil urease activities were determined by the phenol colorimetry of sodium hypochlorite [37]. The soil triphenyl tetrazolium chloride method was used to measure soil dehydrogenase activity [37]. Protease activities were measured by the ninhydrin colorimetric method [37]. The phenol colorimetric method was used to measure soil polyphenol oxidase [37]. The fluorescein diacetate hydrolysis activities were determined by the fluorescein colorimetric method [37].

Soil heavy metals (Cu, Cd, Pb, Zn, and Cr) were measured via a microwave digestion–atomic spectrophotometer (TANK Basic, Hanon, China). First, soil samples were digested using microwave digestion under a mixture of HNO_3_, HCl, and HF (5:2:2, v/v). Then the digestion solution was measured by the atomic absorption spectrophotometer (TAS-990, PGENERAL, Beijing, China) [33].

We calculated the pollution load index (PLI) to evaluate heavy metal contamination at each site, using Equations (1) and (2):

(1) $$CF_{mi}=\frac{Concentration\;of\;each\;heavy\;metal}{Natural\;background\;value}$$

(2) $$PLI_{i}=\sqrt[{5}]{CF_{Cui}\times CF_{Cdi}\times CF_{Pbi}\times CF_{Zni}\times CF_{Cri}{}_{\;}}$$

In Equation (1), *CF* refers to the contamination factor; *m* contains Cu, Cd, Pb, Zn, and Cr, respectively, while *i* represents each study site. In Equation (2), when *PLI* is <1, there is no pollution; when *PLI* is ≥1, but <2, there is low pollution; when *PLI* is ≥2, but <3, there is medium pollution; when *PLI* is ≥3, there is high pollution [38]. According to the Chinese Environmental Quality Standard for Soils (GB 15618-1995), the acceptable contamination levels (mg·kg^−1^) are: Cu 35, Cd 0.2, Pb 35, Zn 100, Cr 90 [39].

### 2.3. DNA Extraction, PCR Amplification, and Illumina MiSeq Sequencing

According to the manufacturer’s instructions, we used the FastDNA™ SPIN Kit for Soil (MP 112 Biomedicals, Solon, OH, USA) to extract DNA from 0.5 g fresh soil samples. The quality of the DNA extracted was examined by 1% (w/v) agarose gel electrophoresis. Additionally, the concentrations were measured by using a UV–vis spectrophotometer (NanoDrop 2000, Thermoscientific, Waltham, MA, USA). The V3–V4 regions of 16S rRNA genes of the bacteria were amplified using the primer set 338F (5′-ACTCCTACGGGAGGCAGCAG-3′) and 806R (5′-GGACTACHVGGGTWTCTAAT-3′). Amplification conditions are as follows: 2 min at 95 °C, 30 cycles of 95 °C for 30 s, 55 °C for 30 s, 72 °C for 30 s, and a final elongation for 5 min in a GeneAmp^®^ 9700 Thermo Cycler (ABI, Foster City, CA, USA). The triplicate amplicons were pooled together, electrophoresed on a 2% (w/v) agarose gel, and recovered through an AxyPrep DNA Gel Extraction Kit (AXYGEN, Hangzhou, China). This purified amplicon was quantified using a QuantiFluor^TM^-ST Fluorometer (Promega, Madison, WI, USA). Then a composite sequencing library was constructed by combining equimolar ratios of amplicons from all the 17 samples. Finally, the sample libraries were analyzed by using the Illumina Miseq platform (Majorbio Bio-Pharm Technology Co., Ltd., Shanghai, China) [35].

### 2.4. Bioinformatics and Statistical Analysis

After Illumina MiSeq sequencing, the original double-ended sequencing data was obtained, which needed to be controlled and filtered by Trimmomatic [40]. After extracting the non-repetitive sequences and removing the single unduplicated sequences, operational taxonomic units (OTU) were clustered at 97% similarity using USEARCH [41]. RDP classifier [42] was used to classify the species. These species’ abundance bar plots were drawn according to the taxonomic results. Mothur software (University of Michigan, Ann Arbor, MI, USA) [43] was used to calculate the Chao1 estimator [44] and the Shannon diversity index [45]. Chao1 (the Chao1 estimator) and Shannon (the Shannon diversity index) reflect the richness and uniformity of a single sample (a specific region or ecosystem) community. The beta diversity distance matrix was calculated using Qiime based on the Bray–Curtis algorithm [41], which is the ratio between regional and local species diversity [46]. The non-metric multidimensional scaling (NMDS) index was analyzed by using the R vegan package, whose aim is to collapse information from multiple dimensions into just a few for better visualized and interpreted [47]. The redundancy analysis (RDA) was analyzed with the R vegan package to detect the relationship between environmental factors, samples, and bacterial flora [35]. The heatmap was used to explore the Pearson relationship between the bacterial community and heavy metals with the R pheatmap package [48]. Duncan’s test was performed by SPSS 22 software (IBM, Shanghai, China), which were applied in the case of multiple comparisons [35]. Charts and graphs were performed using Origin 9.1 (OriginLab, Northampton, MA, USA) or the R project (R Development Core Team, Vienna, Austria) [49].

## 3. Results

### 3.1. Effect of Secondary Lead Smelting on Soil Physicochemical Properties and Enzyme Activities

Soil physicochemical properties are fundamental indicators of soil quality (Table 1). Soil pH values ranged from 6.89 to 7.72, with a mean value of 7.30. Soil pH values near the plant were lower than the control group, indicating that the soil has a certain degree of acidification. Soil organic matter was between 1.06% and 4.11% with a mean value of 2.37%. All 16 samples were lower than the control group. The contents of available potassium ranged from 141.77 to 249.44 mg·kg^−1^ with the mean value of 202.26 mg·kg^−1^, which was higher than the control group. Furthermore, the average concentration of available potassium was high enough to support seasonal crops. The contents of available phosphorus ranged from 4.50 to 58.37 mg·kg^−1^ with an average of 14.58 mg·kg^−1^. While the distribution of available phosphorus had a wide span, it did not show regularity. The mean value of soil nitrate nitrogen was 4.94 mg·kg^−1^ with a range from 1.04 to 13.58 mg·kg^−1^, which was higher than the control. However, although the nitrogen content differed from site to site, each site was enriched in nitrogen such that it provided sufficient concentration for plant growth (Table 1).

Five enzyme activities surrounding the secondary lead plant were determined (Table 1). Soil urease is the critical enzyme in the conversion of nitrogen in the soil and can be measured as a proxy for the status of soil nitrogen. It ranged from 0.3 to 0.61 mg·g^−1^·d^−1^, which presented a higher activity than the control group, 0.27 mg·g^−1^·d^−1^. Soil protease activity ranged from 0.64 and 0.91 mg·g^−1^·d^−1^, which was much higher than the control, 0.18 mg·g^−1^·d^−1^. Soil dehydrogenase, which exercises tight control on respiratory metabolism, ranged from 0.48 to 1.05 g·g^−1^·h^−1^. Soil FDA hydrolytic enzyme, which is widely used in soil quality assessments, ranged from 0.12 to 0.80 mg·kg^−1^·h^−1^. Finally, soil polyphenol oxidase enzyme, which could promote oxidation of phenols, ranged from 0.01 to 0.29 mg·g^−1^·h^−1^. None of the 16 sampling sites around the plant showed any difference from the control soil for these three enzymes (Table 1).

### 3.2. Soil Heavy Metal Concentrations and Containment Assessment near the Secondary Lead Plant

Soil heavy metal contents were shown in Table 2. The concentration of all five heavy metals exceeded the acceptable contamination levels [39]. The heavy metal that occupied the first place was Cd with a peak concentration of 6.97 mg·kg^−1^ at S2, which was 24 times than its background limit. Pb was in the second place with a peak concentration of 223.76 mg·kg^−1^ at S15, surpassing its background value 13 times. Even at their lowest concentrations, Cd and Pb were nearly five times more abundant than their corresponding background values. Cu was the third most abundant heavy metal with peak concentrations of 127.03 mg·kg^−1^ and 123.13 mg·kg^−1^ at S13 and S14, respectively; both of which exceeded its background value 10 times. The concentrations of Zn and Cr did not exceed their background values. The highest concentration of Zn was 203.66 mg·kg^−1^ in S16, while Cr was 153.93 mg·kg^−1^ in S11.

Furthermore, the pollution load index (PLI), used for the detailed evaluation of the degree of soil heavy metal pollution surrounding the secondary lead plant, changed from 1.87 (S8) to 3.69 (S15), with a mean value of 2.69. According to the PLI, 2 sites (S6, S8) showed low heavy metal pollution, 12 sites (S1–S5, S7, S10–S13, S16) showed medium heavy metal pollution, while 4 sites (S9, S14, S15) showed high heavy metal pollution.

### 3.3. Effect of Secondary Lead Smelting on Soil Bacterial Community Diversity

Soil bacterial community diversity was assessed using high-throughput DNA sequencing technology. A total of 591,763 sequences were obtained from 16 soil samples. The length distributions of trimmed sequences ranged from 401 to 600 bp. Under the OTU (operational taxonomic units) at 97% similarity, all rarefaction curves were apt to approximate the saturation platform, which indicated the sequence reads were reasonable [50].

The Chao1 estimator and the Shannon diversity index are shown in Table 3. The Chao1 estimator was at the peak with a maximum index of 2484.27 in S15, and at a minimum of 1763.92 in S1. The Shannon diversity index varied from 6.02 to 6.68. However, the two indices were both lower than the control group. Compared with the control site, the Chao1 estimator decreased about 50%, while the Shannon diversity index dropped approximately 20%.

**Figure 2:**
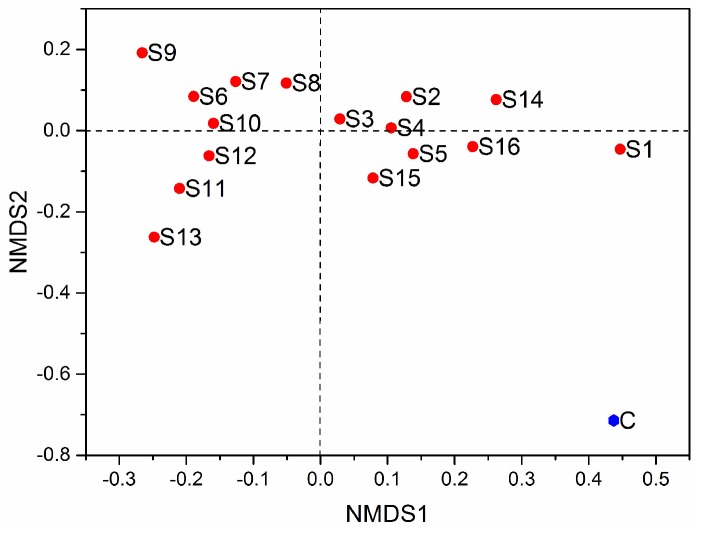
Non-metric multidimensional scaling (NMDS) index analysis of the soil bacterial community near the secondary lead plant.

The non-metric multidimensional scaling (NMDS) index is shown in Figure 2. According to the species information contained in the sample, it is reflected in the multi-dimensional space in the form of points, and the degree of difference between different samples is reflected by the distance between the points and the point, and finally obtains the spatial location map of the sample [51]. The control group and the surrounding sampling sites of the secondary lead plant had a far distance, which announced that the secondary lead smelting activity had severe effects that reduced the soil bacterial diversity in the surrounding soil.

**Figure 3:**
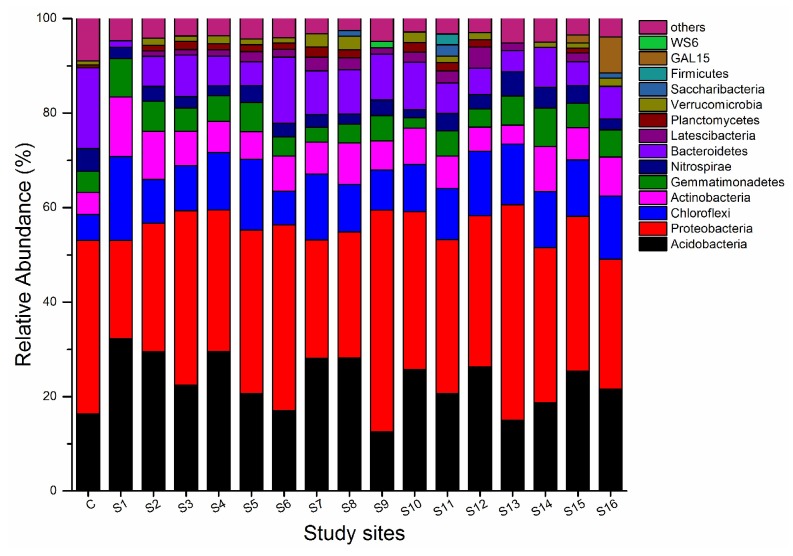
Taxonomic composition of soil samples by phylum near the secondary lead plant.

To further investigate the composition of the bacterial community’s structure, a bar plot of the taxonomic distribution of phyla in soil samples was generated (Figure 3). In the surrounding area of the secondary lead plant, *Proteobacteria*, *Acidobacteria*, *Chloroflexi*, *Actinobacteria*, *Gemmatimonadetes*, *Nitrospirae*, *Bacteroidetes*, *Latrscibacteria*, *Planctomycetes*, *Verrucomicrobia*, *Saccharibacteria*, *Firmicutes*, *GAL15*, and *WS6* occupied more than 90% of the total sequences of each soil sample. The *Proteobacteria* accounted for 20.75–46.90% of the total phyla, while *Acidobacteria* was the second most abundant phylum with a range of 12.57–32.31%; these two bacteria phyla comprised more than half of the bacterial community. Moreover, the relative abundance of *Proteobacteria* was highest in S9 and lowest in S1, while the relative abundance of *Acidobacteria* was greatest in S1 and lowest in S9. Additionally, the relative abundances of *Chloroflexi* and *Bacteroidetes* were greatest at S1 and S6, respectively. Compared with the control group, the relative abundance of *Acidobacteria* and *Chloroflexi* increased, while *Bacteroidetes* and others decreased.

### 3.4. Soil Bacterial Community Response to Changes in Environmental Factors

The correlation between the environmental variables and soil bacterial community matrices could be compared by the Mantel test [52]. In the present study, the soil physicochemical properties, enzyme activities, and heavy metal concentrations of the 16 surrounding areas of the secondary lead plant were divided into three environmental matrices. These groups were then analyzed with the soil bacterial community matrix by the Mantel test. It showed that the soil bacterial community surrounding the secondary lead plant was related to enzyme activities (*r*^2^ = 0.45, *p* = 0.001), and heavy metal contents (*r*^2^ = 0.27, *p* = 0.024), but no clear correlation with soil physicochemical properties (*r^2^ =* −0.13, *p* = 0.391). 

**Figure 4:**
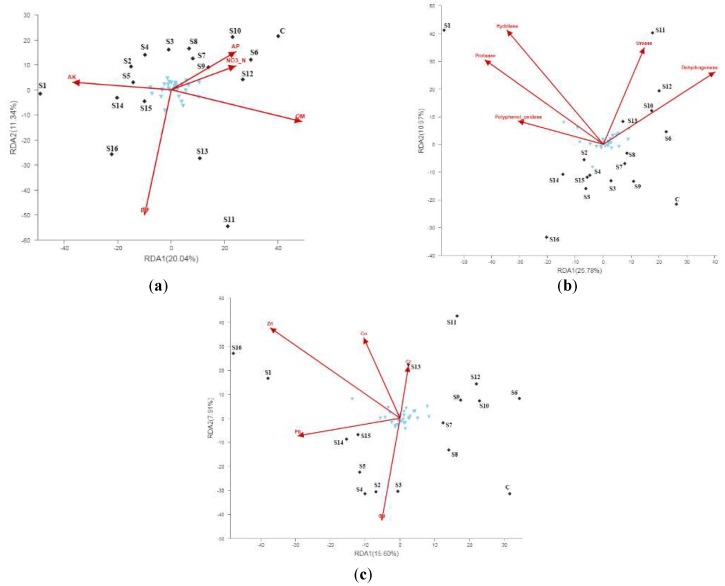
RDA analysis of the bacterial community structure and soil physicochemical properties (**a**), enzymes (**b**), and heavy metals (**c**) near the secondary lead plant. Note: the sky blue inverted triangle is the relative abundance of the top 30 OTUs (operational taxonomic units).

Furthermore, an RDA analysis (R Development Core Team, Vienna, Austria) was conducted to verify the exact effects of physicochemical properties, heavy metals, and enzymes in soil bacterial communities (Figure 4). The results showed that soil pH (*r*^2^ = 0.64, *p* = 0.001) and organic matter (*r*^2^ = 0.58, *p* = 0.008) had a strong positive correlation with soil bacterial community diversity (Figure 4a). The soil enzymes protease (*r*^2^ = 0.72, *p* = 0.003), hydrolase (*r*^2^ = 0.76, *p* = 0.001), and dehydrogenase (*r*^2^ = 0.62, *p* = 0.002) were observed to have a significant effect on soil microbial community diversity (Figure 4b). Moreover, this study found that Zn (*r*^2^ *=* 0.71, *p* = 0.001) and Cd (*r*^2^ = 0.43, *p* = 0.022) had a significant effect on the soil microbial community structure (Figure 4c).

### 3.5. Potential Resistance of Soil Bacteria to Heavy Metals Contamination

**Figure 5:**
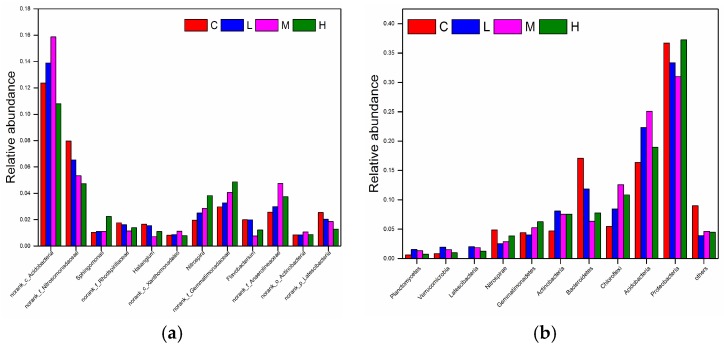
Relative abundance diagram of the pollution and the bacterial by genus (**a**) and phylum (**b**) near the secondary lead plant.

According to the results of the PLI, soil pollution grades near the secondary lead plant could be divided into three groups. The low-pollution group was named L (S6 and S8). The medium-pollution group was named M (S1, S2, S3, S4, S5, S7, S10, S11, S12, S13, and S16); and the high-pollution group was named H (S14, S15, and S9). The unaffected sample was used as the control group (C). The average value of the relative abundance of each group in the genus and the phylum level is shown in Figure 5. As the concentration of heavy metals increased, the relative abundance of *Sphingomonas* and *Nitrospira* increased (Figure 5a). In the presence of higher heavy metal concentrations, *Planctomycetes*, *Verrucomicrobia*, and *Latescibacterial* exhibited lower relative abundances, while the relative abundance of *Nitrospirae* and *Gemmatimonadetes* increased. However, *Actinobacteria*, *Bacteroidetes*, *Chloroflexi*, *Acidobacteria*, and *Proteobacteria*, which dominated the bacterial community, had no apparent correlation with changing heavy metal concentrations. *Actinobacteria* fluctuated with slight decreases as heavy metal concentrations rose. The changes in *Acidobacteria* and *Chloroflexi* were similar, while *Proteobacteria* first declined and then increased (Figure 5b).

**Figure 6:**
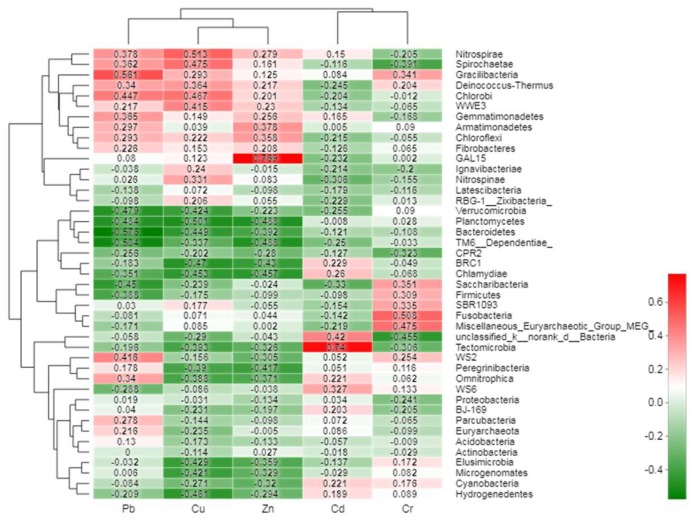
Pearson correlation heatmap between the bacterial distribution of the top 50 abundant phylum and heavy metals near the secondary lead plant. Note: The *x*-axis and *y*-axis of the thermograph are environmental factors and phyla, respectively, and *r*-values and *p*-values are obtained by calculation. The *r*-value is shown in different colors in the graph. The right color card of the thermograph is a color partition with different *r*-values.

Furthermore, the heatmap analysis of each heavy metal with the first 50 bacterial phyla was calculated (Figure 6). The Pb content had negative relationships with both *Bacteroidetes* (*r*^2^ = −0.58, *p* = 0.019) and *TM6 Dependentiae* (*r*^2^ = −0.54, *p* = 0.033), while the decrease of the two bacteria phyla could be used to characterize the increase of Pb pollution. While Pb content and *Gracilibacteria* (*r*^2^ = 0.56, *p* = 0.024) were positively correlated and had a close relationship, Cu and *Planctomycetes* (*r*^2^ = −0.51, *p* = 0.048) were negatively correlated with each other, but were positively correlated with the *Nitrospirae* (*r*^2^ = 0.52, *p* = 0.042). The Zn and *GAL15* (*r*^2^ = −0.77, *p* = 0.001) were negative correlated. Similarly, Cr content had a negative correlation with *Fusobacteria* (*r*^2^ = −0.51, *p* = 0.045). However, no more than two heavy metals could be characterized by a unified group of bacteria.

**Figure 7:**
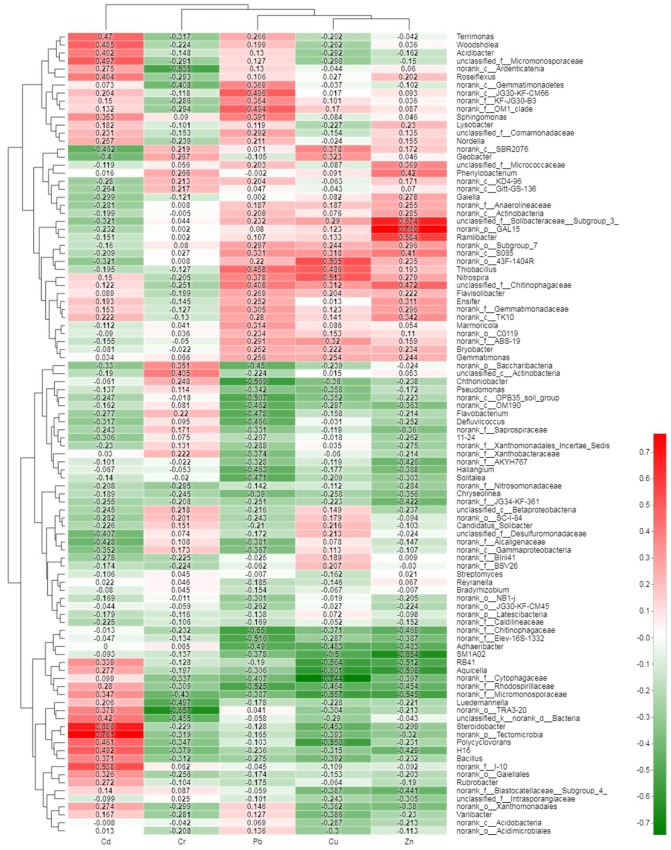
Pearson correlation heatmap between the bacterial distribution of the top 100 abundant genera and heavy metals near the secondary lead plant. Note: The *x*-axis and *y*-axis of the thermograph are environmental factors and phyla, respectively, and *r*-values and *p*-values are obtained by calculation. The *r*-value is shown in different colors in the graph. The right color card of the thermograph is a color partition with different *r*-values.

To identify the impact of heavy metal pollution on the soil bacterial community, the top 100 genera were analyzed through the Pearson correlation heatmap (Figure 7). According to the primary analysis, Zn content had a substantial correlation with *Aquicella* (*r*^2^ = −0.60, *p* = 0.014), *Ramlibacter* (*r*^2^ = 0.58, *p* = 0.018), *RB41* (*r*^2^ = −0.52, *p* = 0.043), *SM1A02* (*r*^2^ = −0.65, *p* = 0.006), and some unclassified or no-rank genus which could belong to *Micromonosporaceae* or *GAL15.* Meanwhile, *Aquicella* (*r*^2^ = −0.61, *p* = 0.023), and SM1A02 (*r*^2^ = −0.5, *p* = 0.049) were negative correlated with Cu content. However, some bacterial genera were strongly correlated with Cu, such as *Nitrospira* (*r^2^* = 0.51, *p* = 0.042) and *Polycyclovorans* (*r*^2^ = 0.52, *p* = 0.025). There was also a correlation between Cd and *Luedemannella* (*r*^2^ = −0.50, *p* = 0.05). The concentration of Cd was significantly correlated with the abundance of *Steroidobacter* (*r*^2^ = 0.69, *p* = 0.003). Finally, it was observed that Cr exhibited a negative correlation with *Luedemannella* (*r*^2^ = −0.50, *p* = 0.006). However, not a single genus was positively correlated with Cr. The bacteria correlating with pollution by specific heavy metals might be an alternative and effective way of forecasting the distribution of heavy metal soil pollution.

## 4. Discussion

We have found that secondary lead smelting activities might cause some changes on the soil physicochemical properties, enzyme activities, heavy metal concentrations, and bacterial communities.

Soil pH, organic matter, available potassium, nitrate nitrogen, and available phosphorus had different changes. According to the pH values, the soil was slightly alkaline, with a mean value of 7.3. It was lower than the background value, ranging from 7.8 to 8.5 [53], which means that secondary lead smelting activities led to soil acidification [54]. This might be due to the emission of sulfur dioxide in the waste smoke and the discharge of acidic waste water during the process of dismantling lead–acid batteries [4,6]. Soil organic matter in all 16 samples is also lower than the control group, which is similar to previous studies [2,4,26]. It covers all four levels of soil organic matter standards that are defined in the second soil survey of Xuzhou [53]. These very large gaps of soil organic matter in different groups also showed the complexity of soil degradation under Pb contamination [54]. At same time, there is a trend of potassium and nitrogen accumulation surrounding the secondary lead plant. Unfortunately, available phosphorus does not show regularity. The result is similar to the previous research conclusion [55,56].

Some soil enzyme activities changed near the secondary lead smelting. Soil urease and protease activity appeared to be enhanced across the whole sampling site. Three other enzymes had no similar changes. This might contribute to the close relationship between urease, protease, and nitrogen cycling [57,58,59].

Secondary lead smelting might cause serious heavy metal contamination [4,12,24,26]. In our study, results have shown that all sites were contaminated, with most sites exhibiting medium or high pollution with heavy metals. Similar to this, industrial smelting activities—such as secondary copper, zinc, and aluminum—have also led to serious heavy metal pollution [60,61,62]. In addition, heavy metal concentrations in the eastern sampling sites, S14 and S15, were higher than other sites, which might be because the eastern sampling sites were near the wastewater outlet of the secondary lead plant [4].

Furthermore, soil bacterial community diversity and composition was reduced near the secondary lead plant. Other studies also reported the decreasing trend in the similar lead smelting activities [2,3,8,21].

This study also found that pH, organic matter, protease, hydrolase, dehydrogenase, Zn, and Cd had a close relationship with the soil bacterial community. Previous studies have yielded both similar and dissimilar results. For instance, one study found that there were no obvious relationships between microbial diversity and soil physicochemical properties [52]. However, another study observed that soil microorganisms were significantly correlated with changes of physicochemical properties [63]. These conflicting results in the previously-mentioned studies might be due to different environmental factors measured in different studies and situations. Setting a pH gradient ranging from 4.0 to 8.3 in an arable soil demonstrated that soil pH has a strong positive influence on soil microbial community structure [64]. Soil organic matter significantly affected the composition and diversity of microbial communities [54]. Additionally, some studies on soil enzyme activity indicated that soil enzyme activity was the primary elements of soil microbial metabolism [65,66,67]. For example, they found that rhizobia has a close relationship with protease and dehydrogenase [66], while proteolytic bacterial communities with showed a close relationship with protease [67].

In addition, some studies reported that heavy metal pollution did not have a simple linear relationship with microbial community structure and composition, which was similar with our results [68,69]. For example, Dmitri and Begonia found that low and medium concentrations of heavy metals promoted microbial diversity, while it would have negatively correlated with microbial richness when heavy metal concentration increased to a certain extent [70]. Doelman and Haanstra claimed long-term heavy metal pollution has a negative effect on soil microbial diversity [71]. Moreover, some studies have reported the complex relationships between specific heavy metal concentrations and bacteria communities. One study on heavy metal pollution in agricultural land observed that aerobic heterotrophic bacteria had a strong positive correlation with Zn [72]. Another found that Cu has little influence on major microbial communities in long-term exposure to heavy metal pollution [73]. Other extensive experiments have shown that Cd stress affected microbial population structure and diversity [74,75,76].

Similar to previous studies in the phylum of relative abundance, *Proteobacteria* was also observed to be the most dominant phylum in the heavy metal contaminated soil [77,78]. This might be due to *Proteobacteria*’s unique biodegradable metabolism and ability to adapt to a wide range of habitats [79]. Some soil bacterial communities with potential resistance to heavy metals might be used for the bio-indicator of metal-polluted soil [80]. In this study, the *Ramlibacter* that were observed might be utilized to predict Zn contamination, while *Nitrospira* and *Polycyclovorans* could be used to diagnose Cu contamination, and *Steroidobacter* could be used to forecast Cd contamination. Judging from previous studies, *Rhodobacter* [81], *Cyanobacteria* [82,83], and *Steroidobacter* [52] might also prove useful to monitor soil heavy metal contamination in the future [83,84]. However, the functions and structures of these newly-discovered bacterial genera need further experimentation.

## 5. Conclusions

In this study, soil physicochemical properties, enzyme activities, heavy metal concentrations, and bacterial diversity near a secondary lead plant of Xuzhou were measured. We conclude the following points:

Firstly, secondary lead smelting altered the physicochemical properties and enzyme activities. Soil acidification decreased one order of magnitude, with a mean value of 7.3. Soil organic matter also showed a downward trend, while potassium and nitrogen appeared to accumulate. Soil urease and protease activity increased in samples with heavier heavy metal pollution. The concentrations of Cu, Cd, Pb, Zn, and Cr exceed the Chinese Environmental Quality Standard for Soils (GB 15618-1995). Moreover, PLI—with a mean value of 2.69—confirmed that heavy metal contamination, especially Pb and Cd, was severe near the secondary lead plant.

Secondly, *Proteobacteria*, *Acidobacteria*, *Chloroflexi*, *Actinobacteria*, *Bacteroidetes*, *Gemmatimonadetes*, *Nitrospirae*, *Latrscibacteria*, V*errucomicrobia*, and *Planctomycetes* comprised more than 90% of the total bacterial community. The most abundant phylum in the soil was *Proteobacteria*, which accounted for 20.75–46.90% of the bacterial community. Furthermore, *Acidobacteria* was the next most abundant phylum, with a range of 12.57–32.31% of the bacterial community. Comparing with the control site, soil microbial diversity decreased, as shown by the decrease of about 50% in the Chao1 estimator, while the Shannon diversity index decreased by approximately 20%.

In addition, soil pH, organic matter, enzyme activities, and the concentration of Zn and Cd had significant correlations with soil bacterial community diversity. Also, several genera were observed to be significantly correlated with heavy metal concentrations. *Ramlibacter*, *Nitrospira* or *Polycyclovorans*, and *Steroidobacter* were strongly correlated with Pb, Cu, and Cd, respectively. These genera might act as bio-indicators for soil remediation, but the function and structure of them will need testing in future studies.

It is anticipated that the results of this research will provide new insight in the future for reclaiming soil contamination caused by secondary lead smelting.

## Figures and Tables

**Table 1:** Soil physicochemical properties and enzyme activities near the secondary lead plant.

Site	Soil Physicochemical Properties					Soil Enzyme Activity				
	pH	SOM ^a^	AK ^b^	AP ^c^	NO_3_^−^-N ^d^	Urease ^e^	Protease ^f^	Dehydrolase ^g^	FDA Dehydrogenase ^h^	Polyphenol Oxidase ^i^
		(%)	(mg·kg^−1^)	(mg·kg^−1^)	(mg·kg^−1^)	(mg·g^−1^·d^−1^)	(mg·g^−1^·d^−1^)	(g·g^−1^·h^−1^)	(g·kg^−1^·h^−1^)	(mg·g^−1^·h^−1^)
C ^j^	8.26i	4.07m	163.9c	14.09k	1.06a	0.27a	0.18a	0.59c	0.23c	0.07f
S1	7.26ef	1.27b	232.1i	5.05b	2.66d	0.49j	0.91j	1.05j	0.26d	0.29l
S2	7.32f	1.79e	249.44k	7.11e	3.86g	0.61o	0.74g	0.64ef	0.53i	0.28k
S3	6.89a	1.53d	141.77a	22.33n	13.58n	0.3b	0.64b	0.63de	0.49g	0.09g
S4	7.03bc	1.76e	173.23d	35.8o	9.69m	0.41f	0.71ef	0.64ef	0.63m	0.28k
S5	7.28ef	1.06a	247.02k	6.71d	2.35c	0.42g	0.71ef	0.65f	0.26d	0.1h
S6	7.13cd	3.28k	174.84d	9.03g	4.75h	0.57l	0.7de	0.59c	0.59l	0.15i
S7	7.27ef	4.11m	240.16j	16.01m	6.4k	0.49j	0.72f	0.54b	0.56j	0.02b
S8	7.18de	2.54i	198.23f	12.24j	3.47e	0.58m	0.74g	0.62d	0.57k	0.03c
S9	6.99ab	2.54i	165.56c	10.51h	3.61f	0.33c	0.66c	0.48a	0.52h	0.01a
S10	6.89a	3.07j	184.92e	11.4i	5.46i	0.59n	0.69d	0.74h	0.47f	0.21j
S11	7.72h	3.31k	147.02b	6.48d	6.92l	0.58m	0.74g	0.74h	0.8o	0.01a
S12	7.58g	2.44h	182.9e	58.37p	5.94j	0.46h	0.72f	0.71g	0.69n	0.01a
S13	7.48g	3.78l	226.05h	14.77l	6.92l	0.55k	0.76h	0.53b	0.49g	0.04d
S14	7.57g	1.42c	234.52i	4.5a	1.32b	0.48i	0.84i	0.7g	0.38e	0.15i
S15	7.48g	1.91f	234.11i	5.38c	1.09a	0.35e	0.71ef	0.76i	0.12a	0.03c
S16	7.71h	2.12g	204.27g	7.51f	1.04a	0.34d	0.71ef	0.53b	0.18b	0.06e

Note: ^a^ SOM: soil organic matter (%); ^b^ AK: available potassium (mg·kg^−1^); ^c^ AP: available phosphorus (mg·kg^−1^); ^d^ NO_3_^−^-N: soil nitrate nitrogen (mg·kg^−1^); ^e^ Urease: soil urease activities (mg·g^−1^·d^−1^); ^f^ Protease: soil protease activities (mg·g^−1^·d^−1^); ^g^ Dehydrolase: soil dehydrolase activities (g·g^−1^·h^−1^); ^h^ FDA: soil FDA dehydrogenase activities (g·kg^−1^·h^−1^); ^i^ Polyphenol oxidase: soil polyphenol oxidase activities (mg·g^−1^·h^−1^); ^j^ C: control site; Values in the same column followed by different small alphabetical letters are significantly different at *p* < 0.05 level based on Duncan analysis (*n* = 3).

**Table 2:** Concentration of heavy metal and pollution load index near the secondary lead plant.

Study Site	Cu (mg·kg^−1^)	Cd (mg·kg^−1^)	Pb (mg·kg^−1^)	Zn (mg·kg^−1^)	Cr (mg·kg^−1^)	PLI ^a^
C	43.7a	0.37a	24.1a	74.3a	47.3a	0.91a
S1	103.62j	1.86c	164.64f	134.52h	111.95f	2.93j
S2	60.69e	6.97i	125.23d	94.32d	83.96d	2.86i
S3	60.69e	4.18g	144.94e	95.12d	69.97c	2.57ef
S4	52.89c	2.79e	164.64f	76.63b	125.94g	2.54e
S5	48.98b	6.04h	105.53c	106.38e	55.98b	2.43d
S6	56.79d	2.32d	85.82b	79.85c	69.97c	1.96c
S7	84.11g	2.32d	85.82b	112.81f	139.93h	2.61f
S8	56.79d	1.86c	85.82b	79.85c	69.97c	1.87b
S9	91.91i	4.18g	105.53c	117.63g	139.93h	3.14l
S10	80.21f	1.39b	105.53c	112.01f	139.93h	2.43d
S11	80.23f	1.86c	85.82b	117.63g	153.93i	2.54e
S12	119.23l	1.86c	164.64f	139.34i	69.97c	2.76h
S13	127.03n	1.86c	184.35g	141.75j	83.96d	2.98k
S14	123.13m	2.32d	164.64f	145.77k	97.96e	3.14l
S15	111.42k	3.25f	223.76h	132.91h	139.93h	3.69m
S16	88.01h	1.39b	125.23d	203.66l	97.96e	2.68g

Note: ^a^ PLI: pollution load index; Values in the same column followed by different small alphabetical letters are significantly different at the *p* < 0.05 level based on Duncan analysis (*n* = 3).

**Table 3:** Chao1 estimator and the Shannon diversity index analysis of the soil bacterial community in contaminated lane in Xuzhou, China.

Study Sites	Chao1 Estimator	Shannon Diversity
C	3060.79h	8.87j
S1	1763.92a	6.01a
S2	2190.08cde	6.49efgh
S3	2406.76ef	6.55fghi
S4	2369.26ef	6.64hi
S5	2328.9def	6.60ghi
S6	2040.8bc	6.19abc
S7	2181.89cde	6.40defg
S8	2108.03bcd	6.32bcde
S9	1984.25bc	6.18abc
S10	2118.81bcd	6.23bcd
S11	2194.34cde	6.29bcde
S12	2305.61def	6.46efgh
S13	1909.72ab	6.14ab
S14	2043.28bc	6.53fghi
S15	2484.26g	6.68i
S16	2314.3def	6.38cdef

Note: Values in the same column followed by different small alphabetical letters are significantly different at *p* < 0.05 level based on Duncan analysis (*n* = 3).

## References

[1] Pidatala V.R., Li K., Sarkar D., Ramakrishna W., Datta R. (2016). Identification of biochemical pathways associated with lead tolerance and detoxification in *Chrysopogon zizanioides* L. Nash (vetiver) by metabolic profiling. Environ. Sci. Technol..

[2] Thornton I., Rautiu R., Brush S. (2001). Lead-the Facts.

[3] Zhang W., Yang J., Wu X., Hu Y., Yu W., Wang J., Dong J., Li M., Liang S., Hu J. (2016). A critical review on secondary lead recycling technology and its prospect. Renew. Sustain. Energy Rev..

[4] Harrison R. (2012). Lead Pollution: Causes and Control.

[5] Ellis T.W., Mirza A.H. (2010). The refining of secondary lead for use in advanced lead–acid batteries. J. Power Sources.

[6] Tian X., Gong Y., Wu Y., Agyeiwaa A., Zuo T. (2014). Management of used lead acid battery in China: Secondary lead industry progress, policies and problems. Resour. Conserv. Recycl..

[7] Indexfuture (2016). Analysis on the Development Status of China’s Renewable Lead Industry in 2016. http://www.indexfuture.net/zklf/228.html.

[8] Bisessar S. (1982). Effect of heavy metals on microorganisms in soils near a secondary lead smelter. Water Air Soil Pollut..

[9] Farago M.E., Thornton I., White N.D., Tell I., Mårtensson M.B. (1999). Environmental impacts of a secondary lead smelter in Landskrona, southern Sweden. Environ. Geochem. Health.

[10] Mao J.S., Cao J., Graedel T.E. (2009). Losses to the environment from the multilevel cycle of anthropogenic lead. Environ. Pollut..

[11] Schneider A.R., Cancès B., Ponthieu M., Sobanska S., Benedetti M.F., Pourret O., Conreux A., Calandra I., Martinet B., Morvan X. (2016). Lead distribution in soils impacted by a secondary lead smelter: Experimental and modelling approaches. Sci. Total Environ..

[12] Liu G., Yu Y., Hou J., Xue W., Liu X., Liu Y., Wang W., Alsaedi A., Hayat T., Liu Z. (2014). An ecological risk assessment of heavy metal pollution of the agricultural ecosystem near a lead–acid battery factory. Ecol. Indic..

[13] Chen K., Huang L., Yan B., Li H., Sun H., Bi J. (2014). Effect of lead pollution control on environmental and childhood blood lead level in Nantong, China: An interventional study. Environ. Sci. Technol..

[14] Cheng H., Hu Y. (2010). Lead (Pb) isotopic fingerprinting and its applications in lead pollution studies in China: A review. Environ. Pollut..

[15] Settle D., Patterson C. (1980). Lead in albacore: Guide to lead pollution in Americans. Science.

[16] WHO WHO/Europe. Environment and Health—Lead Poisoning Prevention Week: Ban Lead Paint. http://www.euro.who.int/en/health-topics/environment-and-health/pages/news/news/2016/10/lead-poisoning-prevention-week-ban-lead-paint.

[17] WHO Childhood Lead Poisoning. http://www.who.int/ceh/publications/leadguidance.pdf.

[18] Šmirjákova S., Ondrašovičová O., Kašková A., Lakticova K. (2005). The effect of cadmium and lead pollution on human and animal health. Folia Vet..

[19] Pan S., Lin L., Zeng F., Zhang J., Dong G., Yang B., Jing Y., Chen S., Zhang G., Yu Z. (2018). Effects of lead, cadmium, arsenic, and mercury co-exposure on children’s intelligence quotient in an industrialized area of southern China. Environ. Pollut..

[20] Brunekreef B., Veenstra S.J., Biersteker K., Boleij J.S.M. (1981). The Arnhem lead study: I. Lead uptake by 1- to 3-year-old children living in the vicinity of a secondary lead smelter in Arnhem, The Netherlands. Environ. Res..

[21] Pattee O.H., Pain D.J. (2003). Lead in the environment. Handb. Ecotoxicol..

[22] De Freitas C.U., De Capitani E.M., Gouveia N., Simonetti M.H., de Paula e Silva M.R., Kira C.S., Sakuma A.M., de Fátima Henriques Carvalho M., Duran M.C., Tiglea P. (2007). Lead exposure in an urban community: Investigation of risk factors and assessment of the impact of lead abatement measures. Environ. Res..

[23] Zhang F., Liu Y., Zhang H., Ban Y., Wang J., Liu J., Zhong L., Chen X., Zhu B. (2016). Investigation and evaluation of children’s blood lead levels around a lead battery factory and influencing factors. Int. J. Environ. Res. Public Health.

[24] Rieuwerts J., Farago M. (1996). Heavy metal pollution in the vicinity of a secondary lead smelter in the Czech Republic. Appl. Geochem..

[25] Luo C., Liu C., Wang Y., Liu X., Li F., Zhang G., Li X. (2011). Heavy metal contamination in soils and vegetables near an e-waste processing site, south China. J. Hazard. Mater..

[26] Gottesfeld P., Were F.H., Adogame L., Gharbi S., San D., Nota M.M., Kuepouo G. (2018). Soil contamination from lead battery manufacturing and recycling in seven African countries. Environ. Res..

[27] Gottesfeld P., Pokhrel A.K. (2011). Review: Lead exposure in battery manufacturing and recycling in developing countries and among children in nearby communities. J. Occup. Environ. Hyg..

[28] Daniell W.E., Van Tung L., Wallace R.M., Havens D.J., Karr C.J., Bich Diep N., Croteau G.A., Beaudet N.J., Duy Bao N. (2015). Childhood lead exposure from battery recycling in Vietnam. BioMed Res. Int..

[29] Chen F., Zhang W., Ma J., Yang Y., Zhang S., Chen R. (2017). Experimental study on the effects of underground CO_2_ leakage on soil microbial consortia. Int. J. Greenh. Gas Control.

[30] Chen F., Tan M., Ma J., Zhang S., Li G., Qu J. (2016). Efficient remediation of PAH-metal co-contaminated soil using microbial-plant combination: A greenhouse study. J. Hazard. Mater..

[31] Chen F., Yang B., Ma J., Qu J., Liu G. (2016). Decontamination of electronic waste-polluted soil by ultrasound-assisted soil washing. Environ. Sci. Pollut. Res..

[32] Chen F., Luo Z., Liu G., Yang Y., Zhang S., Ma J. (2017). Remediation of electronic waste polluted soil using a combination of persulfate oxidation and chemical washing. J. Environ. Manag..

[33] Bao S.D. (2000). Soil and Agricultural Chemistry Analysis.

[34] Chen F., Luo Z., Ma J., Zeng S., Yang Y., Zhang S. (2018). Interaction of cadmium and polycyclic aromatic hydrocarbons in co-contaminated soil. Water Air Soil Pollut..

[35] Ma J., Zhang W., Zhang S., Zhu Q., Feng Q., Chen F. (2017). Short-term effects of CO_2_ leakage on the soil bacterial community in a simulated gas leakage scenario. PeerJ.

[36] Norman R.J., Edberg J.C., Stucki J.W. (1985). Determination of nitrate in soil extracts by dual-wavelength ultraviolet spectrophotometry 1. Soil Sci. Soc. Am. J..

[37] Guan S. (1986). Soil Enzyme and Its Research Methods.

[38] Ahmadi M., Jorfi S., Azarmansuri A., Jaafarzadeh N., Mahvi A.H., Darvishi Cheshmeh Soltani R., Akbari H., Akhbarizadeh R. (2017). Zoning of heavy metal concentrations including Cd, Pb and As in agricultural soils of Aghili plain, Khuzestan province, Iran. Data Brief.

[39] SEPAC (State Environmental Protection Agency of China) (1996). GB15618-1995: Environmental Quality Standard for Soils.

[40] Bolger A.M., Lohse M., Usadel B. (2014). Trimmomatic: A flexible trimmer for Illumina sequence data. Bioinformatics.

[41] USEARCH. http://www.drive5.com/usearch/.

[42] RDP Resources. http://rdp.cme.msu.edu/misc/resources.jsp.

[43] Mothur. https://www.mothur.org/.

[44] Chao A. (1984). Nonparametric estimation of the number of classes in a population. Scand. J. Stat..

[45] Shannon C.E. (2001). A mathematical theory of communication. ACM SIGMOBILE Mob. Comput. Commun. Rev..

[46] Whittaker R.H. (1960). Vegetation of the Siskiyou mountains, Oregon and California. Ecol. Monogr..

[47] Da C Jesus E., Marsh T.L., Tiedje J.M., de S Moreira F.M. (2009). Changes in land use alter the structure of bacterial communities in Western Amazon soils. ISME J..

[48] Udikovic-Kolic N., Wichmann F., Broderick N.A., Handelsman J. (2014). Bloom of resident antibiotic-resistant bacteria in soil following manure fertilization. Proc. Natl. Acad. Sci. USA.

[49] Chang W. (2012). R Graphics Cookbook: Practical Recipes for Visualizing Data.

[50] QIIME. http://qiime.org/.

[51] Noval Rivas M., Burton O.T., Wise P., Zhang Y.-Q., Hobson S.A., Garcia Lloret M., Chehoud C., Kuczynski J., DeSantis T., Warrington J. (2013). A microbiota signature associated with experimental food allergy promotes allergic sensitization and anaphylaxis. J. Allergy Clin. Immunol..

[52] Hong C., Si Y., Xing Y., Li Y. (2015). Illumina miseq sequencing investigation on the contrasting soil bacterial community structures in different iron mining areas. Environ. Sci. Pollut. Res..

[53] SSCO (1985). Soil Chronicles in Suburban Xuzhou City, Jiangsu Province.

[54] Tian J., Lou Y., Gao Y., Fang H., Liu S., Xu M., Blagodatskaya E., Kuzyakov Y. (2017). Response of soil organic matter fractions and composition of microbial community to long-term organic and mineral fertilization. Biol. Fertil. Soils.

[55] Karimi Nezhad M.T., Tabatabaii S.M., Gholami A. (2015). Geochemical assessment of steel smelter-impacted urban soils, Ahvaz, Iran. J. Geochem. Explor..

[56] Shen F., Liao R., Ali A., Mahar A., Guo D., Li R., Xining S., Awasthi M.K., Wang Q., Zhang Z. (2017). Spatial distribution and risk assessment of heavy metals in soil near a Pb/Zn smelter in Feng county, China. Ecotoxicol. Environ. Saf..

[57] Karaca A., Cetin S.C., Turgay O.C., Kizilkaya R. (2010). Effects of heavy metals on soil enzyme activities. Soil Heavy Metals.

[58] Tate R.L. (2002). Microbiology and enzymology of carbon and nitrogen cycling. Enzymes Environment, Activity, Ecology and Applications.

[59] Caldwell B.A. (2005). Enzyme activities as a component of soil biodiversity: A review. Pedobiologia.

[60] Ba T., Zheng M., Zhang B., Liu W., Xiao K., Zhang L. (2009). Estimation and characterization of PCDD/Fs and dioxin-like PCBS from secondary copper and aluminum metallurgies in China. Chemosphere.

[61] Yin X., Yao C., Song J., Li Z., Zhang C., Qian W., Bi D., Li C., Teng Y., Wu L. (2009). Mercury contamination in vicinity of secondary copper smelters in Fuyang, Zhejiang province, China: Levels and contamination in topsoils. Environ. Pollut..

[62] Kuo S.-C., Hsieh L.-Y., Tsai C.-H., Tsai Y.I. (2007). Characterization of PM_2.5_ fugitive metal in the workplaces and the surrounding environment of a secondary aluminum smelter. Atmos. Environ..

[63] Li R., Tao R., Ling N., Chu G. (2017). Chemical, organic and bio-fertilizer management practices effect on soil physicochemical property and antagonistic bacteria abundance of a cotton field: Implications for soil biological quality. Soil Tillage Res..

[64] Rousk J., Bååth E., Brookes P.C., Lauber C.L., Lozupone C., Caporaso J.G., Knight R., Fierer N. (2010). Soil bacterial and fungal communities across a pH gradient in an arable soil. ISME J..

[65] De la Paz Jimenez M., de la Horra A., Pruzzo L., Palma M.R. (2002). Soil quality: A new index based on microbiological and biochemical parameters. Biol. Fertil. Soils.

[66] Rodríguez-Caballero G., Caravaca F., Alguacil M.M., Fernández-López M., Fernández-González A.J., Roldán A. (2017). Striking alterations in the soil bacterial community structure and functioning of the biological N cycle induced by *Pennisetum setaceum* invasion in a semiarid environment. Soil Biol. Biochem..

[67] Wang S., Cheng X. (2017). Changes in proteolytic bacteria in paddy soils in response to organic management. Acta Agric. Scand. Sect. B Soil Plant Sci..

[68] Xie X.-H., Fan F.-X., Yuan X.-W., Zhu W.-X., Liu N., Ping J., Liu J.-S. (2012). Impact on microbial diversity of heavy metal pollution in soils near dexing copper mine tailings. Weishengwuxue Tongbao.

[69] Jiang Y.M., Zhang C., Huang X.L., Ni C.Y., Wang J.F., Song P.F., Zhang Z.B. (2016). Effect of heavy metals in the sediment of Poyang Lake estuary on microbial communities structure base on Mi-seq sequencing. China Environ. Sci..

[70] Sobolev D., Begonia M. (2008). Effects of heavy metal contamination upon soil microbes: Lead-induced changes in general and denitrifying microbial communities as evidenced by molecular markers. Int. J. Environ. Res. Public Health.

[71] Doelman P., Haanstra L. (1984). Short-term and long-term effects of cadmium, chromium, copper, nickel, lead and zinc on soil microbial respiration in relation to abiotic soil factors. Plant Soil.

[72] Kouchou A., Rais N., Elsass F., Duplay J., Fahli N., Ghachtouli N. (2017). Effects of long-term heavy metals contamination on soil microbial characteristics in calcareous agricultural lands (Saiss plain, North Morocco). J. Mater. Environ. Sci..

[73] Narendrula-Kotha R., Nkongolo K.K. (2017). Bacterial and fungal community structure and diversity in a mining region under long-term metal exposure revealed by metagenomics sequencing. Ecol. Genet. Genom..

[74] Gremion F., Chatzinotas A., Kaufmann K., von Sigler W., Harms H. (2004). Impacts of heavy metal contamination and phytoremediation on a microbial community during a twelve-month microcosm experiment. FEMS Microbiol. Ecol..

[75] Duan X., Min H. (2005). Physiological toxicity of Cd^2+^ to representative microbial species in submerged paddy soil. Ecol. Environ.-Ment. Sci..

[76] Li J., Mu Y. (2008). Research advances on the microbial effects of cadmium polluted soil. Environ. Sci. Manag..

[77] Feris K., Ramsey P., Frazar C., Moore J.N., Gannon J.E., Holben W.E. (2003). Differences in hyporheic-zone microbial community structure along a heavy-metal contamination gradient. Appl. Environ. Microbiol..

[78] Gillan D.C., Danis B., Pernet P., Joly G., Dubois P. (2005). Structure of sediment-associated microbial communities along a heavy-metal contamination gradient in the marine environment. Appl. Environ. Microbiol..

[79] Sinkko H., Lukkari K., Sihvonen L.M., Sivonen K., Leivuori M., Rantanen M., Paulin L., Lyra C. (2013). Bacteria contribute to sediment nutrient release and reflect progressed eutrophication-driven hypoxia in an organic-rich continental sea. PLoS ONE.

[80] Hayat R., Ali S., Amara U., Khalid R., Ahmed I. (2010). Soil beneficial bacteria and their role in plant growth promotion: A review. Ann. Microbiol..

[81] Mishra A., Malik A. (2013). Recent advances in microbial metal bioaccumulation. Crit. Rev. Environ. Sci. Technol..

[82] Pereira S., Zille A., Micheletti E., Moradas-Ferreira P., De Philippis R., Tamagnini P. (2009). Complexity of cyanobacterial exopolysaccharides: Composition, structures, inducing factors and putative genes involved in their biosynthesis and assembly. FEMS Microbiol. Rev..

[83] Pereira S., Micheletti E., Zille A., Santos A., Moradas-Ferreira P., Tamagnini P., De Philippis R. (2011). Using extracellular polymeric substances (EPS)-producing cyanobacteria for the bioremediation of heavy metals: Do cations compete for the EPS functional groups and also accumulate inside the cell?. Microbiology.

[84] Sessitsch A., Kuffner M., Kidd P., Vangronsveld J., Wenzel W.W., Fallmann K., Puschenreiter M. (2013). The role of plant-associated bacteria in the mobilization and phytoextraction of trace elements in contaminated soils. Soil Biol. Biochem..

